# MTB-3, a Microtubule Plus-End Tracking Protein (+TIP) of *Neurospora crassa*

**DOI:** 10.1371/journal.pone.0070655

**Published:** 2013-08-12

**Authors:** Rosa R. Mouriño-Pérez, Lorena P. Linacre-Rojas, Ariana I. Román-Gavilanes, Thomas K. Lew, Olga A. Callejas-Negrete, Robert W. Roberson, Michael Freitag

**Affiliations:** 1 Departamento de Microbiología, Centro de Investigación Científica y de Educación Superior de Ensenada, Ensenada, Baja California, México; 2 Department of Biochemistry and Biophysics, Oregon State University, Corvallis, Oregon, United States of America; 3 School of Life Sciences, Arizona State University, Tempe, Arizona, United States of America; Simon Fraser University, Canada

## Abstract

The microtubule (MT) “plus end” constitutes the platform for the accumulation of a structurally and functionally diverse group of proteins, collectively called “MT plus-end tracking proteins” (+TIPs). +TIPs control MT dynamics and link MTs to diverse sub-cellular structures. *Neurospora crassa* MicroTubule Binding protein-3 (MTB-3) is the homolog of yeast EB1, a highly conserved +TIP. To address the function of MTB-3, we examined strains with *mtb-3* deletions, and we tagged MTB-3 with GFP to assess its dynamic behavior. MTB-3-GFP was present as comet-like structures distributed more or less homogeneously within the hyphal cytoplasm, and moving mainly towards the apex at speeds up to 4× faster than the normal hyphal elongation rates. MTB-3-GFP comets were present in all developmental stages, but were most abundant in mature hyphae. MTB-3-GFP comets were observed moving in anterograde and retrograde direction along the hypha. Retrograde movement was also observed as originating from the apical dome. The integrity of the microtubular cytoskeleton affects the presence and dynamics of MTB-3-GFP comets, while actin does not seem to play a role. The size of MTB-3-GFP comets is affected by the absence of dynactin and conventional kinesin. We detected no obvious morphological phenotypes in *Δmtb-3* mutants but there were fewer MTs in Δ*mtb-3*, MTs were less bundled and less organized. Compared to WT, both MT polymerization and depolymerization rates were significantly decreased in Δ*mtb-*3. In summary, the lack of MTB-3 affects overall growth and morphological phenotypes of *N. crassa* only slightly, but deletion of *mtb-3* has strong effect on MT dynamics.

## Introduction

Microtubules (MTs) are part of the cytoskeletal system of all eukaryotic cells and are essential for many vital cellular activities, including maintenance of cell shape, division, migration and intracellular transport [1]–[3]. MTs are “polarized” because they have two distinct ends: a fast-growing, or “plus” end and a slow-growing, or “minus” end. In living cells, the minus-ends are often anchored, whereas the plus-ends are highly dynamic and stochastically switch between phases of growth and shrinkage by addition and loss of α/β tubulin heterodimers, respectively. This process is termed “dynamic instability” and is driven by GTP hydrolysis of the β-tubulin subunits, which is necessary for switching between “catastrophe” (MT depolymerization) and “rescue” (MT polymerization) [4]–[5]. The MT plus-end therefore constitutes a platform for the accumulation of a structurally and functionally diverse group of proteins, collectively called “MT plus-end tracking proteins” (+TIPs). +TIPs control MT dynamics and link MTs to various sub-cellular structures, such as the cell cortex and kinetochores.

“End-binding proteins” (EBs) are core components of +TIP networks due to their autonomous tracking to growing MT ends independently of any known binding partners [6]–[8]. EBs constitute a highly conserved family of proteins, and were initially described as interacting with APC, the adenomatous polyposis coli tumor suppressor protein, which had been implicated in colon cancer [9]. Homologues of EB1 (i.e., EB2, EB3) have been described not only for vertebrates [10]–[12], but also for plants, e.g. AtEB1a in *Arabidopsis thaliana* [13], insects, e.g. DmEB1 in *Drosophila melanogaster* [14], and fungi, e.g. BIM1 in *Saccharomyces cerevisiae* [15], MAL3 in *Schizosaccharomyces pombe* [16], PEB1 in *Ustilago maydis* [17] and AN2862.3 in *Aspergillus nidulans* [18]. EB1 localizes to the mitotic spindle and cytoplasmic MTs, at their distal tips, and regulates MT dynamics promoting anti-catastrophe activity (i.e., MT growth), cell polarization, and chromosome stability [2], [3], [19]. Hence, EB1 has been described as a good marker for the dynamics at the growing tips of MTs because it directly dictates the behavior of the MT plus-ends [20]. EB1 binds autonomously to growing MT ends, where it forms comet-like accumulations and interacts directly with the majority of other known +TIPs, stimulating MTs assembly and consequently their growth [1]–[3], [7], [21]–[25].

Previous studies in *N. crassa* have shown that cytoplasmic MTs are arranged generally longitudinal along the hyphal tube and that they move as a unit as hyphae are extending, although anterograde and retrograde motility have also been described in small MTs [5], [26]–[27]. We expected that the *Neurospora* EB1 homologue can play a significant role in orchestrating MT dynamics. Consequently, the main goal of this work was to characterize the localization and behavior of the *N. crassa* EB1 homologue, MicroTubule Binding protein-3 (MTB-3), during the dynamic instability of MTs, polarized growth and development, as well as the characterization of the MTB-3 deletion mutant.

## Materials and Methods

### Strains and culture conditions

Strains used in this study are listed in Table 1. Strains were maintained on Vogel's minimal medium (VMM) with 2% sucrose. All manipulations were according to standard techniques [28]. We used a ro-3 mutant with an UV-induced point mutation in p150glued, the largest subunit of dynactin [29]–[30], and a Δkin-1 deletion mutant that lacks conventional kinesin (“nkin”; [31]).

**Table 1:** Strains used in this study.

Organism	Genotype	Reference
NC4200	*mat a*	FGSC
NMF39	*mat A*	FGSC2489
N2928	*mat A his-3; Δmus-51::bar* ^+^	FGSC9717
FGSC43	*mat a; ro-3*	FGSC43
N2281-3A	*mat A his-3* ^+^ *::Pccg-1-hH1* ^+^ *-sgfp* ^+^	Freitag *et al.*, 2004
N2524	*rid^RIP1^ mat a his-3* ^+^ *::Pccg-1-Bml* ^+^ *-sgfp* ^+^	Freitag *et al*., 2004
NMF138	*mat A his-3* ^+^ *::Pccg-1-hH1* ^+^ *-tdimerRed* ^+^	Freitag *et al*., 2004
FGSC11851	*mat a; Δmtb-3*	Colot *et al.*, 2006
FGSC11852	*mat A; Δmtb-3*	Colot *et al.*, 2006
XRM-10-OC	*mat a his-3; Δkin-1*	This study
XTL2-1	*mat A his-3* ^+^ *::Pccg-1-mtb-3* ^+^ *-sgfp* ^+^	This study
XTL2-2	*mat a his-3* ^+^ *::Pccg-1-mtb-3* ^+^ *-sgfp* ^+^	This study
XRM-01-AR	*his-3* ^+^ *::Pccg-1-Bml* ^+^ *-sgfp* ^+^ *;Δmtb3*	This study
XRM-02-AR	*his-3* ^+^ *::Pccg-1-hH1* ^+^ *-sgfp* ^+^ *; Δmtb3*	This study
XRM-03-AR	*his-3* ^+^ *::Pccg-1- mtb3* ^+^ *-sgfp* ^+^ *; ro3*	This study
TRM-01-AR	*Δkin-1; MTB-3* ^+^ *::sgfp* ^+^	This study
TRM-02-AR	*mat A his-3* ^+^ *::Pccg-1-mtb3* ^+^ *-mChfp*	This study

### Construction of fps-containing plasmids

Standard PCR and cloning procedures were used to fuse the *sgfp* (green fluorescent protein; GFP) or *mchfp* (mCherry fluorescent protein; mChFP) gene to the carboxyl terminus of *mtb-3*. The *mtb-3* gene (1,019 nt) was amplified by PCR from *N. crassa* genomic DNA isolated from a wild type (WT) strain (NMF39; FGSC2489) with primers that included XbaI and BamHI or PacI restriction endonuclease sites at their respective 5′ termini, the reverse primer eliminated the stop codon of *mtb-3* to generate a translational fusion to *sgfp* or *mChfp* (OMF1, 5′-CCGTCTAGAATGGGTGAATCACGGTGTGTA-3′ and OMF2, 5′- GGCGGATCCTAAAGGTCTCCTGATCGTCGGC-3′) for pTL1; and for pRM43-AR01. PCR products were digested with XbaI and BamHI and cloned into XbaI- and BamHI-digested plasmid pMF272 (GFP, GenBank accession no. AY598428; [26]), or digested with XbaI and PacI and cloned into XbaI- and PacI-digested pJV15-2 (mChFP; [32]). This yielded pTL1 and pRM43-AR01, respectively. Inserts were verified by sequencing.

### Transformation protocols, transformant selection and crosses

Transformation of *N. crassa* strain N2928 (FGSC9717) conidia with linearized plasmids (Table 1) was carried out by electroporation as previously described [33]–[34]. Prototrophic His+ transformants were screened for the expression of GFP or mChFP by epifluorescence microscopy as described previously [26]. Transformants showing fluorescence were selected. Heterokaryotic transformants were backcrossed to *mat a* strains to isolate homokaryotic progeny, following standard protocols [28]. For each cross, recipient strains were grown on medium for 5 days at 25°C and fertilized by adding conidia from the donor strain. After ∼14 days of incubation at 25°C, ascospores from the developed perithecia were collected from Petri dish covers. Ascospores were spread on VMM, heat-shocked at 60°C for 60 min and incubated overnight at room temperature or 32°C. Colonies were transferred to slants with VMM and incubated at 28°C. Strains were screened under an epifluorescence microscope to select progeny that expressed GFP or mChFP.

### Complementation of Δ*mtb-3* by MTB-3-GFP

Strains carrying deletion alleles of *mtb-3* (FGSC11851 and FGSC11852) were obtained from the Neurospora Functional Genomics project from the Fungal Genetics Stock Center (FGSC, Kansas City, MO). They were crossed to XTL2-1 and XTL2-2 to generate homokaryotic strains that carried MTB-3-GFP in the *mtb-3* deletion background, resulting in XRM-AR-01and XRM-AR-02, respectively (Table 1).

### Actin and MT depolymerization assays

Stock solutions of the anti-actin drug cytochalasin A (CytA; CAT# 14110-64-6, Sigma–Aldrich, St. Louis, Mo), and the anti-microtubule drug benomyl (Ben; CAT# PS222, Sigma–Aldrich) at 10 mg ml^−1^ were prepared in 100% ethanol. For further studies the concentration that inhibited the hyphal growth rate by 50% was selected based on our previous work [35]. To study the effect of CytA on the distribution of MTB-3 in *N. crassa*, we exposed the cells to 1 µg ml^−1^ of CytA in VMM plates and incubated at 28°C until the cells reached a young mycelium stage (∼16 h). For Ben experiments, we inoculated VMM plates amended with 2.5 µg ml^−1^ and incubated at 28°C. For both treatments, mycelia were observed following the procedure described for laser scanning confocal microscopy.

### Laser scanning confocal microscopy of living cells

To record the behavior of all the strains under study, we used the “inverted agar block” method [36] for live-cell imaging with an inverted laser scanning microscope (LSM-510 Meta, Carl Zeiss, Göttingen, Germany) equipped with an argon ion laser for excitation at 488 nm wavelength and GFP filters for emission at 515–530 nm, and a He-Ne laser for excitation at 543 nm and mChFP filters for emission at 580–700 nm. An oil immersion objective was used: 100× (PH3)/1.3 N.A. planneofluar. Laser intensity was kept to a minimum (1.5%) to reduce photobleaching and phototoxic effects. Time-lapse imaging was performed at scan intervals of 0.5 to 4.5 s for periods up to 40 min. Image resolution was 512×512 pixels and 300 dpi. Confocal images were captured using LSM-510 software (version 3.2; Carl Zeiss) and evaluated with an LSM 510 Image Examiner. Some of the image series were converted into AVI movies using the same software. Time-lapse images of hyphae were recorded simultaneously by phase contrast microscopy and fluorescent confocal microscopy. Phase contrast images were captured with a photomultiplier for transmitted light using the same laser illumination for fluorescence [27]. Final images were processed, and figures were created using Adobe Photoshop CS3 Extended (Adobe Systems Inc, San Jose, CA). For fluorescence recovery after photobleaching (FRAP), a selected rectangular area of a hypha was overexposed at 20% intensity for 30 s (argon ion laser 488 nm wavelength). After photobleaching, images were scanned at intervals of 3.5 to 4.5 s for up to 3 min [27].

### Total Internal Reflection Fluorescence Microscopy (TIRFM) of living cells

For TIRFM, an IX-70 inverted microscope equipped with a 60×/1.45 N. A. Apochromat objective lens (Olympus Corp., Melville, NY) and a krypton/argon laser (Melles Griot, Carlsbad, CA) (488 nm) was used [37]. Images were recorded with a Cascade 512B EMCCD camera (Photometrics, Tucson, AZ) for durations of 2–3 min at 512×512 resolution and frame rates of 50–100 msec. MetaMorph 6.0/6.1 software (Universal Imaging, Downingtown, PA) was used to control the camera and capture images. Final images were processed, and figures were created using Adobe Photoshop CS3 Extended (Adobe Systems Inc, San Jose, CA).

### Membrane staining with FM4-64

Using the “inverted agar block method” of preparing and staining samples [36], hyphae of GFP-expressing strains were double-labeled with 2.5 µM FM4-64 (Invitrogen, Eugene, OR), which stains the plasma membrane and organelle membranes [26], [38].

## Results

### Localization and dynamics of MTB-3-GFP in *N. crassa*

**Figure 1:**
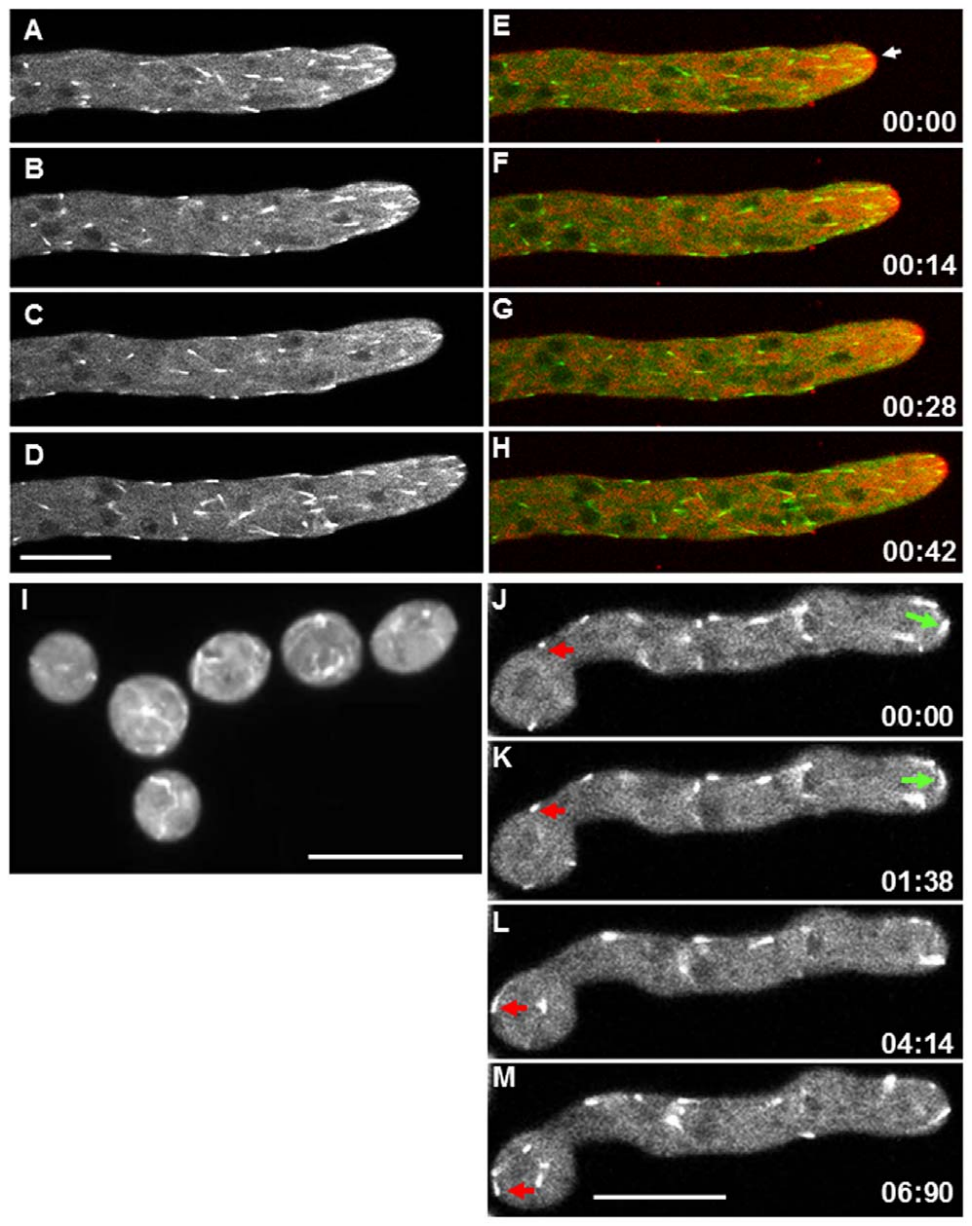
Distribution of MTB-3-GFP comets in different developmental stages of *N. crassa*. (A–D) Time lapse images of MTB-3-GFP in mature hyphae. (E–H) merged images of MTB-3-GFP and membranes stained with 5 µM FM4-64 in mature hypha. The Spitzenkörper is indicated by a white arrow. (I) Conidia expressing MTB-3-GFP. (M–P) Time lapse images of MTB-3-GFP in germlings. Red arrows point to MTB-3-GFP comets that move in retrograde direction and green arrows point to comets that move in anterograde direction. Time scale is in min∶sec. Scale bars = 10 µm.

MTB-3-GFP was present as comet-like structures that appeared evenly distributed along the hypha (Fig. 1A–H; Movie S1). The comets had a length of 1.44±0.03 µm (mean ± standard error; n = 150). MTB-3-GFP comets moved mainly towards the apex with a speed of 0.64±0.02 µm s^−1^, about fourfold faster than the hyphal elongation rate (0.15±0.01 µm s^−1^) (p<0.05). We also observed instances of comets moving in retrograde direction (Movie S1). In distal regions, comets of MTB-3-GFP moved in both anterograde and retrograde directions along the hyphae and accumulated in regions around the nuclei. MTB-3-GFP comets were present in all developmental stages, but were less prevalent in spores (Fig. 1I) and germlings (Fig. 1J–M) than mature hyphae. MTB-3-GFP comets were seen moving in anterograde and retrograde direction along germ tubes and the germinal spore (Fig. 1J–M).

FRAP experiments, corroborated the faster speed of MTB-3-GFP comets relative to the cytoplasmic bulk flow through the growth axis of hyphae. The comets moved faster than the cytoplasm or the elongating tip, thus it was not possible to observe defined bleached areas; after application of the laser, the bleached area was immediately re-populated by fluorescent comets.

**Figure 2:**
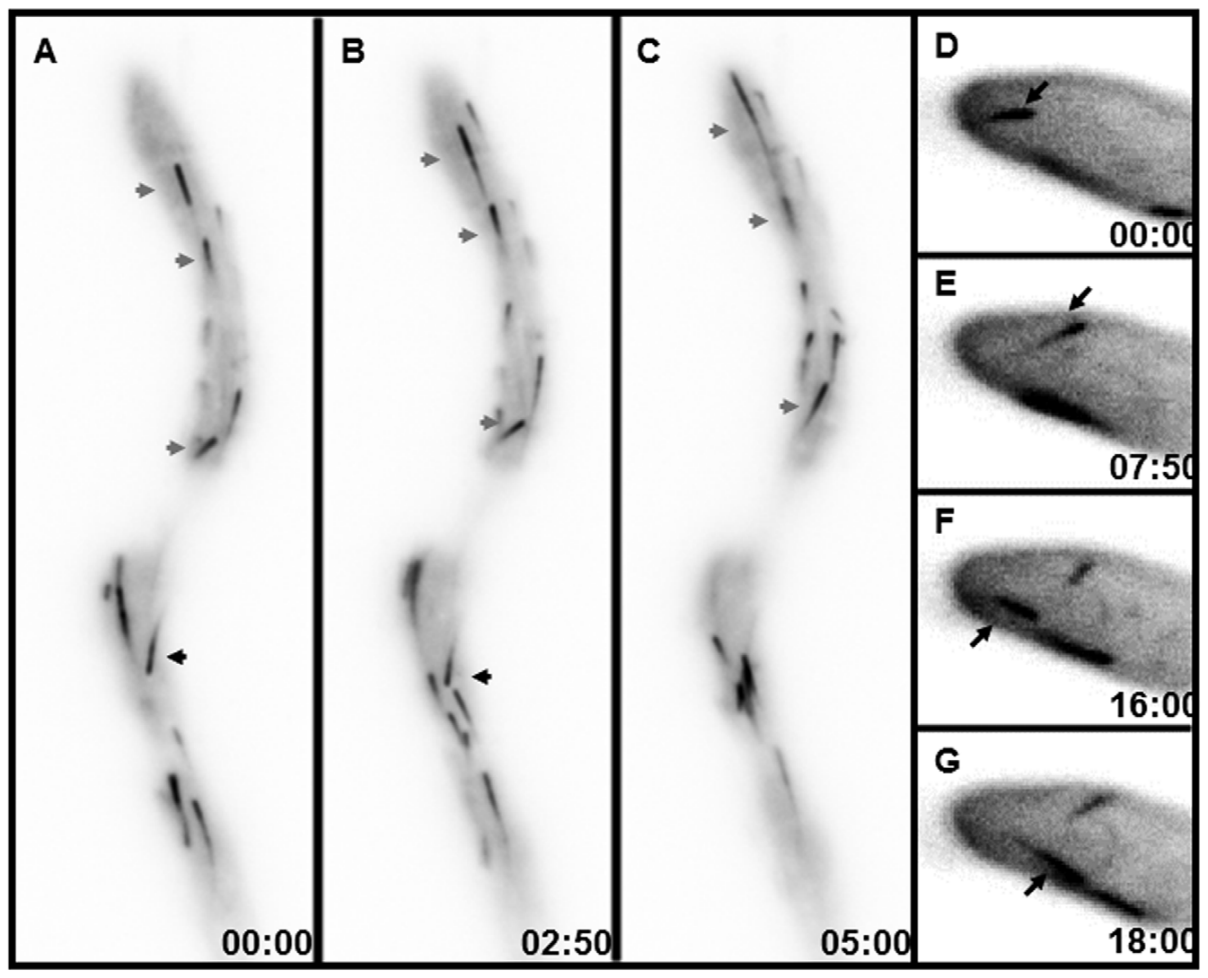
TIRFM of MTB-3-GFP in the cortex of mature hyphae. (A–C) Apical and subapical regions. (D–G) Enlargement of apical regions. Grey arrows point to MTB-3-GFP comets moving in anterograde direction, and black arrows point to comets that move in retrograde direction. Time scale is in sec∶msec. Scale bars = 10 µm.

**Figure 3:**
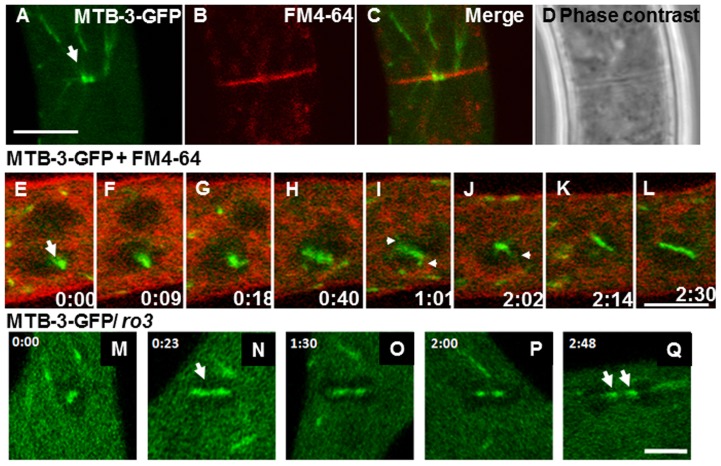
Localization of MTB-3-GFP in different hyphal regions of *N. crassa*. (A–D) MTB-3-GFP remains close to the septal pore. (A). Septum of a mature hypha expressing MTB-3-GFP. (B) Membranes stained FM4-64 (C) Merged image of (A), (B) and (D) Phase contrast image. Arrow points to MTB-3-GFP accumulation in the completely formed septal pore when it is already formed. Scale bar = 10 µm. (E–L) Confocal time lapse images of a nucleus during mitosis in mature hypha of a strain expressing MTB-3-GFP, merged with images of membranes stained with FM4-64. The arrow points to accumulation of MTB-3-GFP on the spindle of a nucleus in mitosis. Arrowheads indicate the presence of MTB-3-GFP in what seem to be astral MTs. Time scale is in min∶sec. Scale bar = 5 µm. (M–Q) Confocal time lapse images of a nucleus during mitosis in a strain expressing MTB-3-GFP in the *ro-3* mutant. Arrows show MTB-3-GFP in the spindle, even when karyokinesis has been completed. Time scale is in min∶sec. Scale bar = 5 µm.

Additionally, both anterograde and retrograde motility of MTB-3 comets were detected in apical and subapical regions of the hyphal cortex when TIRFM was used (Fig. 2; Movie S2). With TIRFM, we were able to observe instances of comets moving from apical to basal regions of the hypha, suggesting the plus-end of MTs were growing in retrograde direction (Fig. 2D–2G). In other regions, MTB-3-GFP accumulations were observed in the septal pore where the MTs are trapped during septation (Fig. 3 A–D). The accumulation of MTB-3-GFP in the mature septa lasted throughout the whole observation period. MTB-3-GFP was also localized along the mitotic spindle during nuclear division. Although it is not possible to determine whether the fluorescence is associated to certain part of the spindle, it seems that MTB-3-GFP is decorating the whole spindle and also part of what seems to be astral MTs (Fig. 3 E–L). The dynamics of MTB-3-GFP in the mitotic spindle was not affected in the dynactin p150^Glued^ subunit, the fluorescence of MTB-3-GFP was present in all mitotic stages and also after karyokinesis (Fig. 3M–3Q).

### MTB-3 during depolymerization of MTs and actin microfilaments

**Figure 4:**
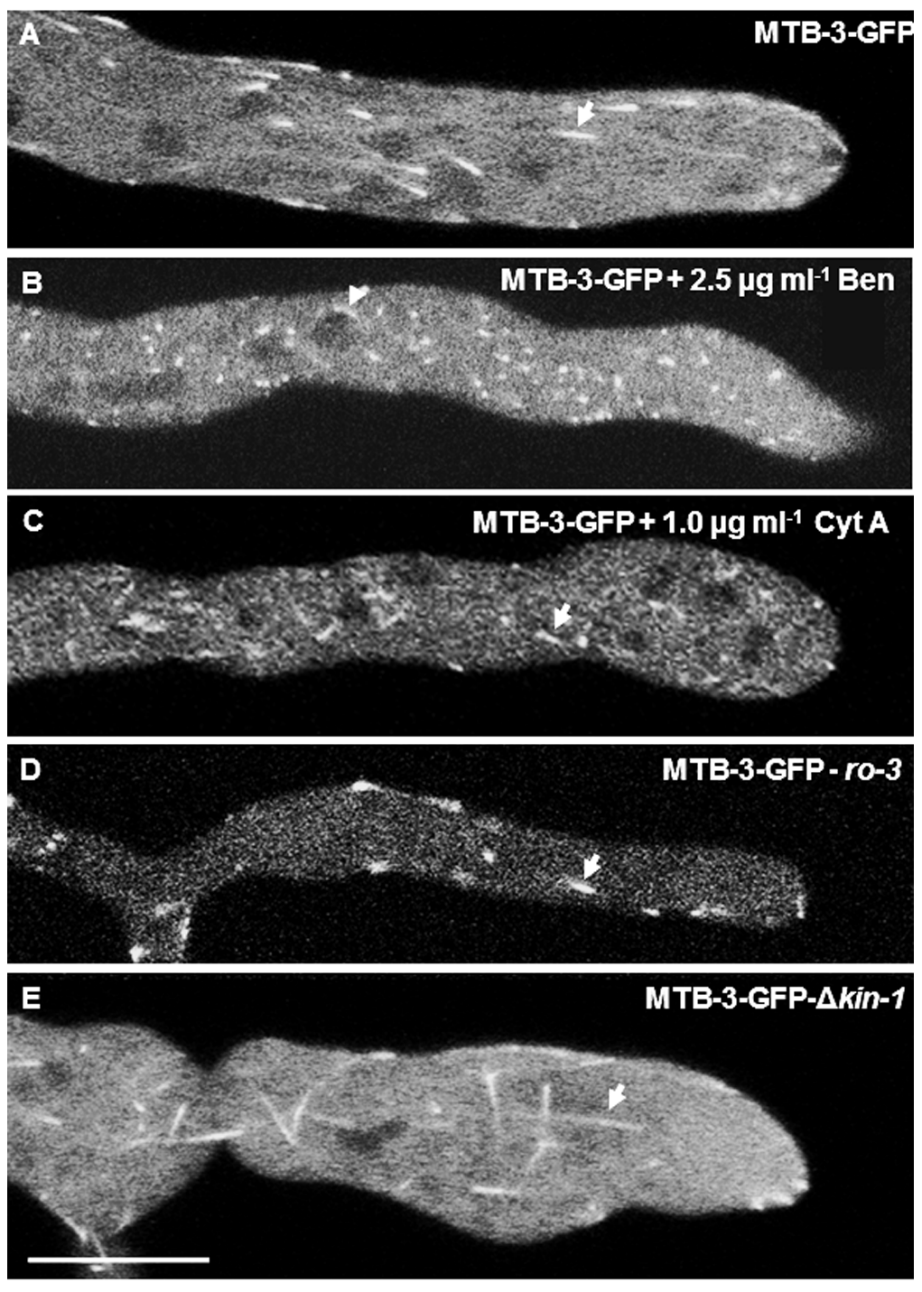
Effect of drugs anti MTs and actin and mutation of motor proteins in MTB-3-GFP comets. Effect of benomyl (Ben), cytochalasin A (CytA) or mutation of motor proteins (ro-3 and Δ*kin-1*) on localization and dynamics of MTB-3-GFP comets. (A) MTB-3-GFP in the WT strain in untreated mature hypha, (B) MTB-3-GFP in mature hypha treated with 2.5 µg ml^−1^ of Ben to depolymerize MTs. (C) MTB-3-GFP in mature hypha treated with 1.0 µg ml^−1^ Cyt A, to disturb the actin cytoskeleton. (D) MTB-3-GFP in the dynactin mutant (p150^glued^ subunit, *ro-3*) and (E) MTB-3-GFP in the conventional kinesin deletion mutant (Δ*kin-1*). Arrows point to the MTB-3-GFP comets in each treatment and mutant. Scale bar = 10 µm.

When MT cytoskeleton was depolymerized with 2.5 µg ml^−1^ Ben [35], all MTB-3-GFP comets disappeared, resulting in a punctuated and dispersal distribution of the fluorescence throughout the hypha (Fig. 4B). Only a few disorganized fluorescent accumulations were present along the hypha and some of them were concentrated around nuclei (Fig. 4B, arrowhead). As MTB-3 has a calponin domain, a motif that can regulate interactions of proteins with actin filaments, we also performed an experiment with the anti-actin drug CytA (1 µg ml^−1^) to observe potential effects of actin filament depolymerization on localization and integrity of MTB-3-GFP comets. We used a concentration of CytA that ensures disruption of the actin cytoskeleton without compromising cell survival [39]. Fluorescent comets were seen in treated hyphae, but they were fewer and smaller than WT strain (Fig. 4C).

### The lack of MT associated motor proteins and MTB-3 dynamics

**Figure 5:**
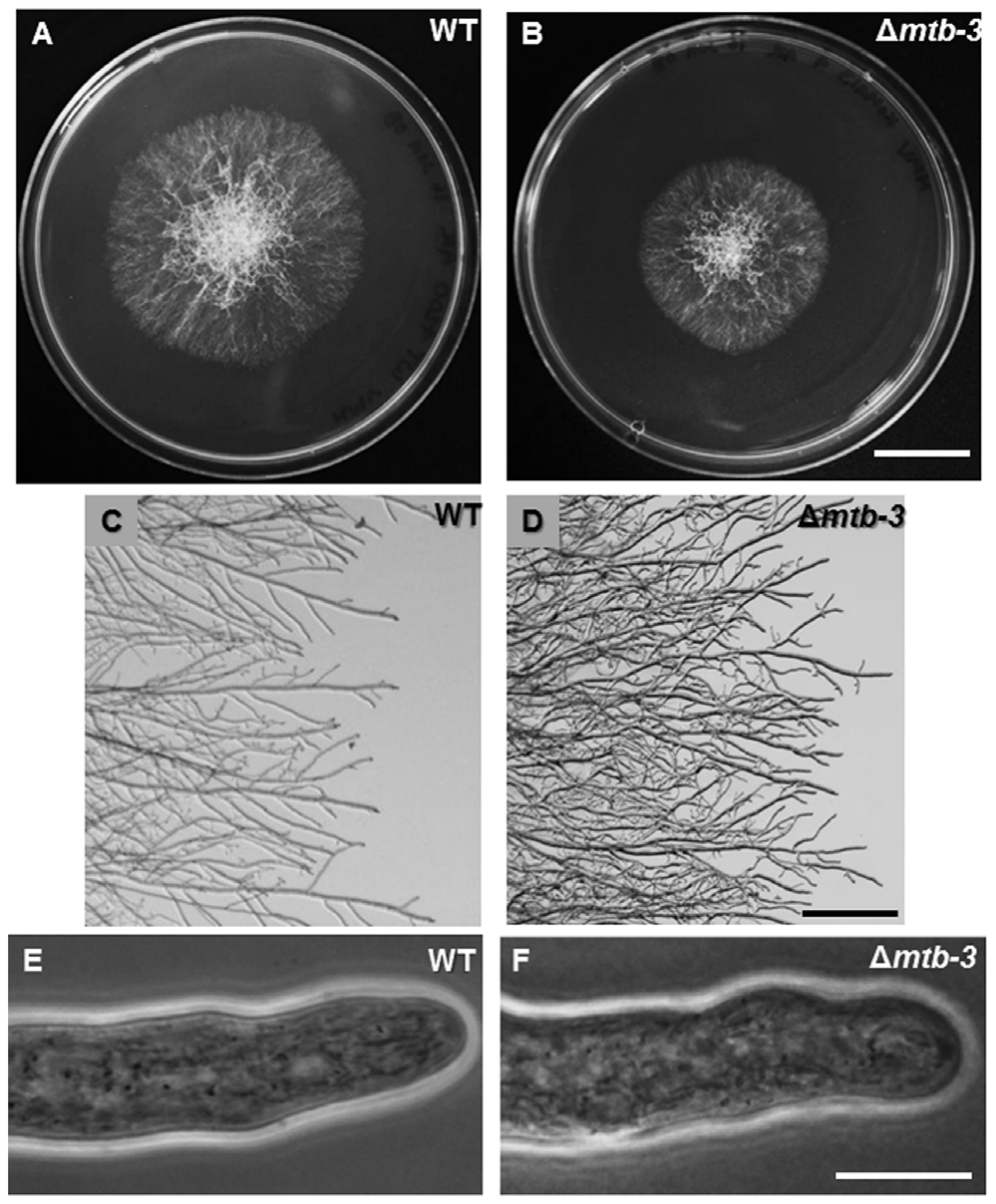
Phenotype of the Δ*mtb-*3 mutant. Macroscopic growth phenotype of colonies of (A) WT and (B) Δ*mtb-3*, showing slightly reduced linear growth of the mutant. Stereomicroscopic image of (C) WT and (D) Δ*mtb-3*, showing more compact colony margins of the mutant. Hyphal phenotype of (E) WT and (F) Δ*mtb-3*, showing slightly more rounded appearance of the apex in the mutant. Scale bar (A–B) = 2 cm, (C–D) = 100 µm and (E–D) = 10 µm.

Irregular sizes of MTB-3-GFP comets were distributed evenly along the hypha of dynactin (*ro*-3) and conventional kinesin (Δ*kin*-1) mutants (Fig. 5D–E). Slightly longer MTB-3-GFP comets were observed in *ro-3* (1.83±0.05 µm; n = 100) relative to WT strains (Table 2) (p<0.05). Also, the abundance of fluorescent comets were reduced and motility was slower (0.42±0.02 μ s−1) than in WT (p<0.05) (Fig. 5D, Movie S3), resulting in altered shapes (blotchy instead of needlelike). In Δ*kin*-1 mutant, MTB-3-GFP comets were two-times longer (3.42±0.10 µm; n = 100) than in WT (p<0.05), but they moved at similar speeds (0.68±0.03 µm s^−1^) to MTB-3-GFP comets as in WT (p>0.05) (Fig. 5E; Table 2; Movie S4).

**Table 2:** Average (± standard error) of length and speed of MTB-3 for WT, Δ*kin-1* and *ro-3* strains.

	WT	Δ*kin-1*	*ro-3*
Length (µm)	1.44±0.03	3.42*±0.10	1.83*±0.05
*n* =	150	100	100
Speed (μ s^−1^)	0.64±0.02	0.68±0.03	0.42*±0.02
*n* =	50	50	50

The number of observations is indicated by *n*.

* p<0.05.

#### Characterization of *mtb-3* deletion strains

**Figure 6:**
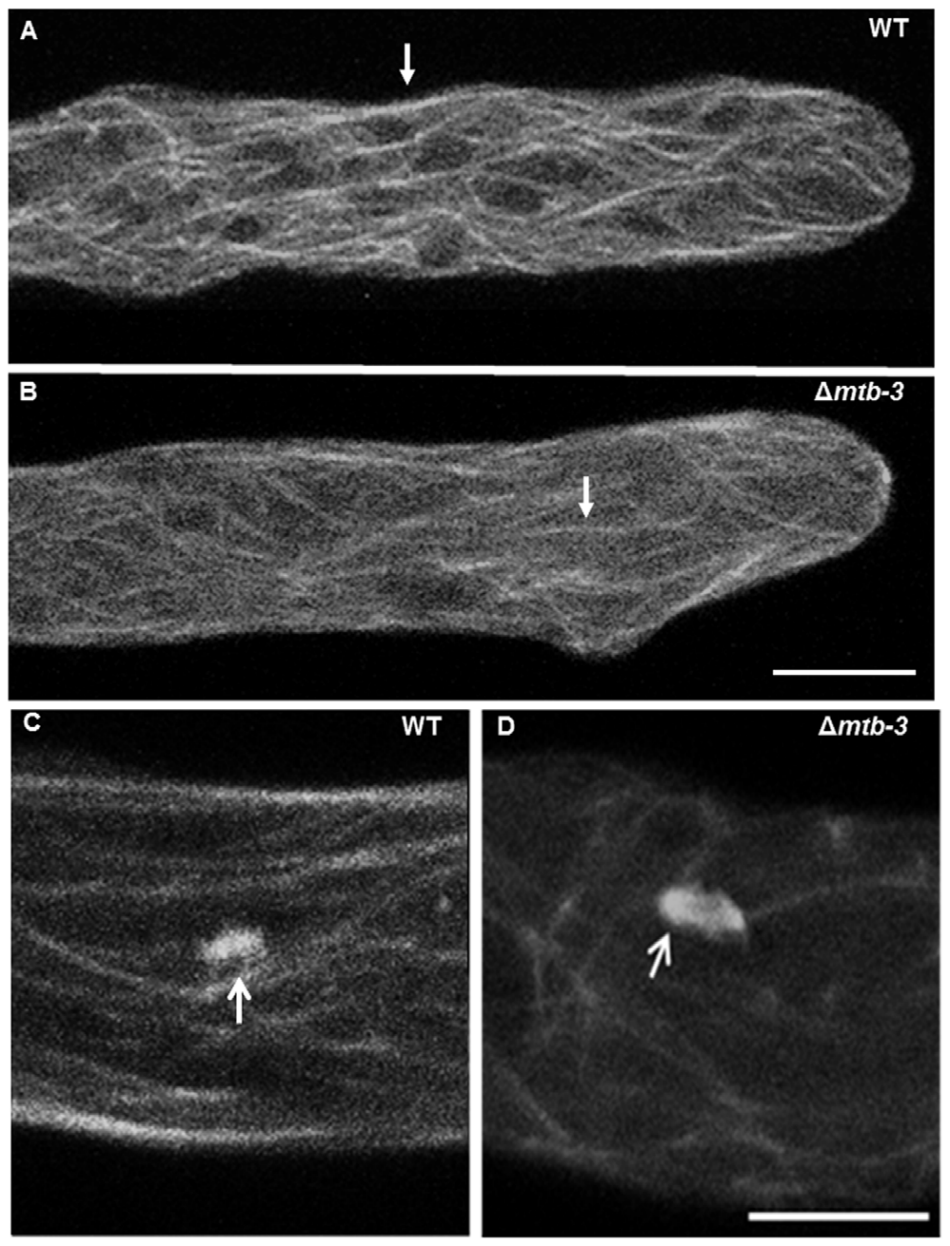
MTs in the Δ*mtb-*3 mutant. MT distribution, measured by imaging of β-tubulin-GFP, and dynamics in mature hyphae of (A) WT and (B) Δ*mtb-3* (also see Movies S5 and S6). The white arrow indicates a thicker and brighter arrangement of MTs, while the red arrow points to thinner appearance of MTs in Δ*mtb-3*. Mitotic spindle in (C) WT and (D) Δ*mtb-3*. It was easily observed how MTs are not compacted in mitotic spindles in Δ*mtb-3* (white arrow) in (D). Scale bar = 5 µm.

We found no obvious, morphological phenotypes associated with deletion of the *mtb-3* gene. We found, however, slightly reduced linear growth rates and slightly increased conidiation rates compared to WT (Fig. 6, Table 3). The linear elongation rate in Δ*mtb-3* mutant was 34.1±1.1 µm min^−1^ compared to 41.9±0.4 µm min^−1^ in WT (p>0.05). Images obtained by stereomicroscopy showed somewhat denser mycelium in Δ*mtb-3* mutant. However, no differences in branching rate or biomass accumulation were observed (Fig. 6 C–D, Table 3).

**Table 3:** Average (± standard error) of elongation, branching, conidiation and biomass rates for WT and Δ*mtb-3*.

	WT	Δ*mtb-3*
Elongation rate (µm s^−1^)	41.9±0.4	34.1±1.1
Branching in 500 lineal-µm	2.0±0.2	2.0±0.2
*Total number of hyphae measured*	45	45
*Total number of branches*	111	84
*Total number of bilateral branches*	20	14
*% Bilateral branching*	18	17
Conidiation rate (conidia ×10^7^ per ml)	86.7±2.5	117.2±5.6
Biomass (mg of dry weight per day)	193.2±2.7	192.9±5.8

### Microtubules dynamics in Δ*mtb-3* mutant

**Figure 7:**
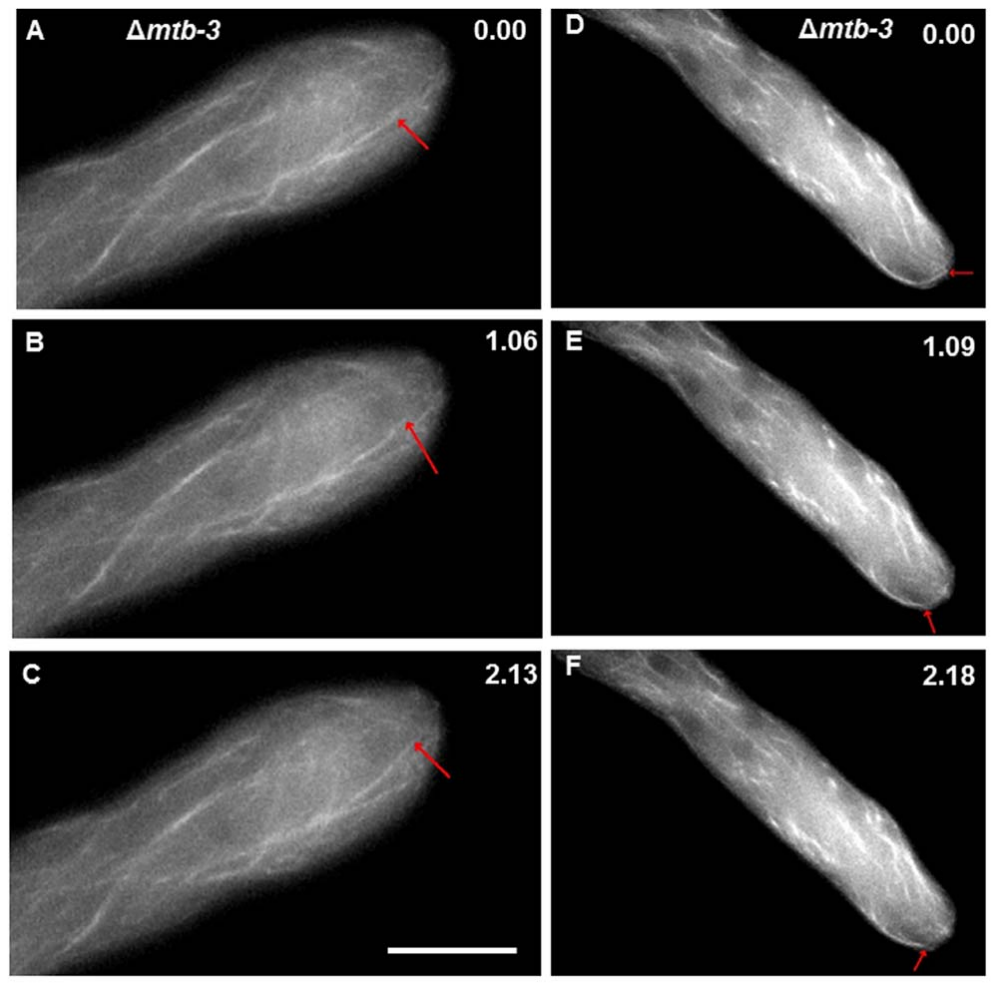
Dynamic instability of MTs in the Δ*mtb-3*. Instances of (A–C) a MT polymerizing and (D–F) depolymerizing in mature hyphae of the Δ*mtb-3*. The MT plus ends are indicated with red arrows. Time scale in min∶sec. Scale bar = 10 µm.

MTs tagged with GFP (β-tubulin-GFP) were observed in Δ*mtb-3* by confocal microscopy compared with WT strain (Fig. 7). MTs in Δ*mtb-3* appeared thinner and less organized in bundles than in WT (Fig. 7B). MTs close to the apex were slightly less organized in Δ*mtb-3* mutant; fewer MTs reached the apical dome relative to WT (Fig. 7; Movie S5). Additionally, spindle MTs were less compact during nuclear division in Δ*mtb-3* compared to WT strain (Fig. 7 C+D). Dynamic instability of MTs appeared to be modified in Δ*mtb-3* mutant, polymerization (21.91±15.72 µm s^−1^) and depolymerization (38.90±26.98 µm s^−1^) rates in Δ*mtb-3* mutant were reduced by 21% and 28%, respectively, relative to WT (Fig. 7, Table 4).

**Table 4:** Average (± standard error) rates of dynamic instability of apical microtubules in mature hyphae of WT and Δ*mtb-3*.

	WT	Δ*mtb-3*
Polymerization rate	27.96±6.34	21.91*±2.49
(µm min^−1^)	*n* = 36	*n* = 40
Depolymerization rate	58.24±6.42	38.90*±4.93
(µm min^−1^)	*n* = 34	*n* = 30

The number of observations is indicated by *n*.

* p<0.05.

## Discussion

### MTB-3-GFP in *N. crassa*

A large variety of +TIPs are localized to the MTs plus-end. These proteins have different domains, structures and functional properties. EB1 homologues are core components of +TIP networks because of their autonomous tracking to growing MT plus ends, which is apparently independent of any known binding partners [7]–[8]. MTB-3 is the single EB1 homologue found in the *N. crassa* genome. MTB-3 tagged with GFP was localized at the plus-end of cytoplasmic and mitotic microtubules as comet-like structures. We also observed MTB-3-GFP in septal rings, where MTs are trapped during septation [26]. MTB-3-GFP comets were also observed in germlings and conidia, but in lower abundance than in mature hyphae.

The fluorescent comets of MTB-3 have been visualized moving not only toward apical but also basal regions, similar to the anterograde and retrograde motility of MTs and nuclei observed previously in *N. crassa* [5], [27]. Observing MT dynamics by autonomous tracking of MTB-3 with two independent microscopic techniques (confocal and TIRF), suggests that the MT plus-ends are growing in both directions along mature hyphae and germlings with speeds about four-times higher than the typical hyphal elongation rate. The direct binding of MTB-3 to the plus-end of polymerizing MTs was also corroborated, when only fluorescent yet stationary spots labeled by MTB-3-GFP were observed after exposure to benomyl at concentrations that depolymerized the microtubular cytoskeleton and altered hyphal shape and growth [35].

Conversely, even though we found homology to a calponin domain in *Neurospora* MTB-3, no evidence of strong disturbance in the MTB-3 dynamics along hyphae was found after cytochalasin A exposure. This was unexpected as calponin domains are often found in actin-binding proteins [40]. Only a slight reduction in the number and size of MTB-3 comets was observed, likely as an indirect consequence of the actin depolymerization that affected MTs at the apical and subapical regions [35]. Our observations of MTs plus-ends growing in both anterograde- and retrograde directions including the apical region, suggested an antiparallel growth of MTs from different nucleation sites distributed within the cytoplasm and likely very close to the apex of *N. crassa* [27], [41]. This behavior has been previously seen in interphase MTs of *U. maydis*; however, in those studies there was no indication for mixed polarities of MTs in the growing region [17], [42].

In spite of the relationship between motor proteins and the dynamic instability of MTs in *N. crassa* [5], we found no evidence for direct interactions of MTB-3 with dynactin and conventional kinesin, suggesting an autonomous behavior of this +TIP in this filamentous fungus. This may be accomplished by binding of MTB-3 to the MT plus-end through recognition of specific plus-end structure, thus stimulating MT assembly (polymerization) and consequently aiding MT growth [2]–[3]. In fact, the model of the interaction of MTB-3 with a certain nucleotide state of β-tubulin (GTP) [43] helps to explain that a lack of conventional kinesin (Δ*kin-1*) in *N. crassa*, which likely caused an increase of GTP-β-tubulin in the microtubular structure, generated MTB-3 comets twice as long as those in WT. However, the displacement dynamic of the MTB-3 comets seemed unaltered by the lack of this motor protein, as shown by the similar speeds of comets in Δ*kin-1* and WT strains. This suggests that MTB-3 can reach MT tips independently. The lack of dynactin in *ro-3* mutant only slightly increased the length of MTB-3 comet while causing appreciable changes in comet shape. We also observed a considerable decrease in the abundance and motility of MTB-3 comets along the mature hyphae in *ro-3*, caused by a reduction in the MT abundance and dynamic instability rates as were previously observed (reduction of 50% and 53% of polymerization and depolymerization rates; [5]). A decrease in the polymerization rate in *ro-3* may cause distortions in “treadmilling”, which will affect directly how much MTB-3 is accumulated on MT tips and how rapidly it is exchanged with the cytoplasmic pool. This can cause an illusion of comets moving along the hypha, as suggested elsewhere [3]. Thus, it is possible that slower treadmilling was observed in *ro-3* due to a reduced association-dissociation rate of MTB-3 with the binding sites at the growing MT, either by a decrease in the structural features at the MTs plus-end or by a low recognition signal of those features.

#### Function of MTB-3 in *N. crassa*

Previous work has suggested that EB1-like proteins specifically decorate freshly polymerized MT plus ends and that this fulfills a conserved role in promoting MT polymerization by increasing MT rescue frequencies and decreasing MT catastrophe or pausing [2], [10], [12], [15]–[16], [19]. Here, we observed a decrease in polymerization and depolymerization rates, an alteration in the form and size of MTs, thus resulting in thinner or less associated MT bundles, and reduced hyphal elongation rates in Δ*mtb-3* strains. Our results suggest an important role of MTB-3 in maintaining integrity of the microtubular cytoskeleton, and therefore in normal growth and development of *N. crassa*. Variations in MT morphology were also observed in Mal3 mutants of the fission yeast, *S. pombe*. Lack of *mal3* resulted in short and often weak cytoplasmic MTs [16]. Maintenance of MT structural integrity by this class of +TIP was also observed in the budding yeast Bim1 homologue of the human pathogen *Cryptococcus neoformans* [44]. Abnormally short and relatively unstructured MTs were observed in *bim1* mutants in filaments during sexual development and during diploid filamentation, consequently changing its normal filamentous growth [44]. Similarly, MT dynamics, specifically during G1 phase of cell division, has been shown on studies of *S. cerevisiae* Bim1 [15].

We also show a role for MTB-3 in keeping the structural integrity of the MT cytoskeleton in mitotic MTs of *N. crassa*. Lack of MTB-3 caused less compact mitotic spindles during nuclear division, probably by a reduction of structural stability of interpolar MTs as part of mitotic spindles. Indeed in previous work, deletion of *S. cerevisiae bim1* showed shortening of the MTs that generated a reduction in the length of the anaphase antiparallel overlap zone, which is important for the efficient antiparallel cross-linking of motor proteins that maintain the forces required to stabilize the elongating anaphase spindle [45]. Additionally, MTs at the start of mitosis switch from polymerization to depolymerization at a rate that is 20 times faster than that of interphase MTs [46]. Therefore, the reduction in the dynamic instability rates by >20% that we found when *Neurospora* MTB-3 was lacking likely altered the initial assembling of the mitotic spindle, generating a more relaxed structure in Δ*mtb-3* than in WT.

The distal segment of MTs has been described as a “plus-end raft” that allows a cascade of proteins interactions that control the dynamics and function of the microtubular cytoskeleton [47]. Particularly, the interaction between EB1 with other proteins to stabilize the MTs during their growth has been reported. For example, interactions between EB1 and dynactin suggest that they function as a plus-end complex. While the p150^Glued^ subunit of dynactin has potent activity for MT nucleation, EB1 has the ability to elongate MTs, thus the two proteins together would have large effects on overall MT dynamics [48]. In our work, deletion of the *N. cassa mtb-3* gene, likely caused a partial loss of this plus-end complex functionality and altered the balance between individual effects of MTB-3 and the network of all other +TIPs on overall microtubule dynamics, suggested by the reduced instability dynamics and hyphae elongation rates observed. Consequently, our findings highlight the importance of MTB-3 not only in maintaining the integrity of the microtubular cytoskeleton structure but also, to interact with other +TIPs at the MTs plus-end, in order to guarantee optimal polarized growth and development of the filamentous fungus *N. crassa*.

## Supporting Information

Movie S1 **MTB-3-GFP comets dynamics in mature hypha of** ***N. crassa*** **.** Comets are distributed homogeneously along the hypha and moved in anterograde and retrograde direction.
(AVI)

Movie S2 **TIRFM of MTB-3-GFP cortical comets dynamics in mature hypha of** ***N. crassa*** **.**
(AVI)

Movie S3 **Dynamics of MTB-3-GFP in the UV mutant of the p150glued subunit of dynactin (** ***ro-3*** **).**
(MPG)

Movie S4 **Dynamics of MTB-3-GFP in the deletion mutant of kin-1 gene (Δ** ***kin-1*** **).**
(MPG)

Movie S5 **Microtubules dynamics (β-tubulin-GFP) in the** ***mtb-3*** **gene deletion mutant.**
(MPG)

Movie S6 **Microtubules dynamics (β-tubulin-GFP) in the WT.**
(MPG)
